# *Epimedium koreanum* Nakai–Induced Liver Injury—A Mechanistic Study Using Untargeted Metabolomics

**DOI:** 10.3389/fphar.2022.934057

**Published:** 2022-07-13

**Authors:** Pin Li, Lin Zhang, Zhaojuan Guo, Qianjun Kang, Cong Chen, Xiaoyao Liu, Quantao Ma, Jingxuan Zhang, Yujie Hu, Ting Wang

**Affiliations:** Beijing Institute of Traditional Chinese Medicine, Beijing University of Chinese Medicine, Beijing, China

**Keywords:** herb-induced liver injury, Epimedium koreanum Nakai, metabolomics, glutathione, ferroptosis

## Abstract

Epimedii Folium is widely used worldwide as an herbal supplement, and the risk of its induced liver damage has emerged in recent years. Our preliminary study has found that, among several Epimedii Folium species specified in the Chinese Pharmacopoeia, *Epimedium koreanum* Nakai has a more severe propensity for hepatotoxicity. However, the mechanism of hepatotoxicity of *Epimedium koreanum* Nakai is still unclear. In this study, untargeted metabolomics was performed to analyze the serum and liver tissue to explore the mechanism of hepatotoxicity of *Epimedium koreanum* Nakai. The results of experiments *in vivo* showed that, after 28 days of exposure to *Epimedium koreanum* Nakai ethanol extract (EEE), the liver weight, levels of AST, ALP, TBIL, etc. in serum of rats in the EEE group were significantly increased, as well as severe cytoplasmic vacuolation appeared in the liver tissue, which suggested that EEE has significant hepatotoxicity. Subsequently, the results of metabolomics revealed significant changes in the metabolic profile in the liver and serum of rats after EEE exposure, in which metabolites in serum such as flavin mononucleotide, phenylacetylglycine, glutathione, l-tryptophan, and sphingomyelin were able to accurately identify liver injury caused by EEE and could be used as serum markers to reflect EEE-induced liver injury. The KEGG pathway enrichment analysis revealed that EEE caused extensive effects on rats' metabolic pathways. Some of the most affected pathways included glutathione metabolism, glutamate metabolism pathway, primary bile acid biosynthesis pathway, and sphingolipid metabolism pathway, which were all directed to the biological process of ferroptosis. Then, the main markers related to ferroptosis in the liver were examined, and the results demonstrated that the content of malondialdehyde was significantly increased, the activity of superoxide dismutase was significantly reduced, the ferroptosis inhibitory proteins GPX4 and System x_c_^−^ were significantly downregulated, and the ferroptosis-promoting protein ACSL4 was significantly up-regulated. Judging from these results, we concluded that the mechanism of hepatotoxicity of *Epimedium koreanum* Nakai was probably related to the induction of ferroptosis in hepatocytes.

## Introduction

Epimedii Folium (Yinyanghuo in Chinese) is the dried leaf of *Epimedium brevicornu* Maxim., *Epimedium sagittatum* (Siebold&Zucc.) Maxim., or *Epimedium pubescens* Maxim. or *Epimedium koreanum* Nakai, in the family berberidaceae (Chinese Pharmacopoeia Commission, 2020). In the theory of Traditional Chinese Medicine, Epimedii Folium has the ability to dispel wind and cold, tonify the kidneys, and strengthen the tendons (Ma et al., 2011). It is widely used to treat various diseases, such as osteoporosis, impotence, infertility, cardiovascular disease, and amnesia (Ma et al., 2011). However, in recent years, two registered drugs with Epimedii Folium as the main ingredient, Zhuangguguanjie Pill and Xianlinggubao Capsules, were reported to be associated with liver injury in humans in clinical applications (Cheng et al., 2013; Tang et al., 2017; Tang et al., 2018; Wu et al., 2019), which has attracted widespread attention. Drug-induced liver injury is one of the most serious and common adverse drug reactions in clinical practice (Navarro et al., 2017), and severe liver injury may induce acute liver failure in patients and even lead to the death of patients (Tujios and Fontana, 2011; Iorga et al., 2017). Therefore, it is important to investigate the potential risk of liver injury of Epimedii Folium. At present, the research on the hepatotoxicity of Epimedii Folium has been gradually carried out. Some researchers have found that Icariside I and Icariside II (the main components of Epimedii Folium) could induce specific hepatotoxicity by enhancing the activation of nlrp3 inflammasome (Wang Z et al., 2020; Gao et al., 2021). Other researchers alsohave found that both 2″-O-rhamnosylicariside II and Sagittatoside B caused severe hepatocyte vacuolation and hepatocyte degeneration in adult zebrafish after 15 consecutive days of treatment (Zhong et al., 2019). However, there are few studies on the mechanism of Epimedii Folium inducing hepatotoxicity *in vivo*.

Most of the liver injury caused by Chinese herbal medicine *in vivo* is extremely complex and shows significant individual differences (García-Cortés et al., 2016; Brown, 2017). Metabolomics reveals the intrinsic mechanisms of growth and development, disease, and environmental influences on the body through a comprehensive and systematic analysis of metabolites in body fluids, tissues, or cells (Gong et al., 2017; Gika et al., 2019), which has been increasingly applied in exploring the mechanism of hepatotoxicity of herbal medicines (Duan et al., 2018). Zhaoyan Zhang et al. found that Polygoni Multiflori Radix extracts could cause liver damage by interfering with *α*-linolenic acid metabolism, taurine and taurine metabolism, glycerophospholipid metabolism, and primary bile acid biosynthetic pathways based on a metabolomic approach (Zhang et al., 2019). Yusha Luo et al. found that gardenia might cause liver injury by disrupting pyrimidine, purine, and amino acid metabolism and pantothenic acid and Coenzyme A biosynthesis through metabolomic methods (Luo et al., 2021). Applying metabolomics to study the hepatotoxicity of Epimedii Folium can help not only to clarify the toxicological characteristics of Epimedii Folium but also to reveal its potential mechanism.

In a previous study, we have found that *Epimedium koreanum* Nakai had more severe hepatotoxicity among several Epimedii Folium prescribed in the Chinese Pharmacopoeia, and its mechanism of hepatotoxicity was considered to be related to oxidative stress by experiments *in vitro* (Zhang L et al., 2020). However, the detailed mechanism of liver injury caused by *Epimedium koreanum* Nakai *in vivo* has not been elucidated. Based on the status of the prophase management research, we planned to study the mechanism of hepatotoxicity of *Epimedium koreanum* Nakai in this study by hepatotoxicity evaluation *in vivo* and non-targeted metabolomics method. The hepatotoxicity of *Epimedium koreanum* Nakai ethanol extract (EEE) was first investigated and validated by histopathological and biochemical methods, and the serum and liver samples were analyzed by untargeted metabolomics. Then, the differential metabolites of these samples were identified and screened using the UPLC-Q-TOF/MS technology platform and were analyzed to predict the pathways of EEE-induced liver injury *in vivo*. Subsequently, the mechanisms were investigated and analyzed by detecting key indicators in the pathway. This study could provide a reference for the safe utilization of Epimedii Folium in clinical practice.

## Materials and Methods

### Chemicals and Reagents

Methanol and acetonitrile (UHPLC grade) were purchased from Fisher Chemical (Darmstadt, Germany). Ammonium acetate (70221) and sodium pentobarbital (P3761) were purchased from Sigma-Aldrich (St. Louis, Missouri, United States). Icaritin (110737-201516, purity >98%) and epimedin C (111780-201503, purity >98%) were purchased from the National Medical Products Administration (Beijing, China). Epimedin A (C15J3G1, purity >98%), epimedin B (C28S3G1, purity >98%), epimedin C (H16D5X1, purity >98%), and baohuoside I (H16D5X1, purity >98%) were purchased from Shanghai yuanye Bio-Technology Co., Ltd. (Shanghai, China). Alkaline phosphatase (ALP, AUZ8511), alanine transaminase (ALT, AUZ9151), aspartate aminotransferase (AST, AUZ9022), direct bilirubin (DBIL, AUZ9056), and total bilirubin (TBIL, AUZ8613) assay kits were purchased from Beckman Coulter (Suzhou, China). The malondialdehyde (MDA, M496) assay kit was purchased from Dongren Chemical Technology Co., Ltd. (Shanghai, China). The superoxide dismutase (SOD, S0101S) activity assay kit was purchased from Beyotime Biotechnology (Shanghai, China). *Epimedium koreanum* Nakai was obtained from the backup samples of previous experiments in our laboratory's herbal sample collection room (Zhang L et al., 2020).

EEE was produced by extracting dried *Epimedium koreanum* Nakai twice with 10 volumes of 70% ethanol-water (V/V), and the extract solution from both times was filtered and concentrated at 50 °C under negative pressure. The concentrates were freeze-dried to obtain a brown extract, and the ratio of the obtained extract from the original herb was 35.2%.

### Quality Assessment of *Epimedium koreanum* Nakai Ethanol Extract

To evaluate the quality of EEE, we determined the concentrations of the five main components in EEE using high-performance liquid chromatography (HPLC). Standard solutions preparation: a mixed standard solution of icaritin, epimedin C, epimedin A, epimedin B, epimedin C, and baohuoside I was prepared using 50% DMSO: H_2_O solution with the concentrations of 11.1 μg/ml, 12.5 μg/ml, 22.9 μg/ml, 12.1 μg/ml, and 9.4 μg/ml for each compound.

Sample solution preparation: 0.5 g EEE was added into 100 ml 50% DMSO: H_2_O solution (V/V) and sonicated for 30 min to dissolve fully. After cooling to room temperature, the solution was filtered using a 0.45 μm membrane to obtain the sample solution.

The HPLC method used for the detection was described in the following way: C_18_ column (Platisil, 4.6 mm × 250 mm, 5 μm, Decima Technology Co., Ltd., Beijing, China). The mobile phase was acetonitrile (A): water (B), and the procedure of gradient elution was (time/A%): 0–25 min, 25%–27%; 25–45 min, 27%–49%; 45–65 min, 49%–81%. The chromatographic analysis was performed with the column temperature maintained at 25°C, mobile phase flow rate at 1 ml/min, detection wavelength at 277 nm, and sample injection volume of 10 µl.

### Animals Handling and Experimental Design

Male Sprague Dawley rats (180–200 g) were purchased from Beijing Vital River Laboratory Animal Technology Co., Ltd (license number SCXK-[jing] 2016–0011). The rats used for the experiments were housed at the Animal Experiment Center of Beijing University of Chinese Medicine, where room temperature was maintained at 20 ± 2°C and humidity at 60–70%, and a 12-h light/dark cycle was maintained. Rats had free access to a standard diet and water throughout the experimental period. All rats were acclimatized to this environment for 4 days before the experiment.

After acclimatization, the experimental rats were randomly divided into control and EEE groups (*n* = 6, each group). EEE was processed as solutions with deionized water before being used. The EEE group was administered EEE solution at 2 g original herb/kg (0.704 g EEE/kg) for 28 days, in a volume of 1 ml/100 g, once a day. The dosage of EEE was based on our previous studies (Zhang et al., 2018). The control group was also given an equal amount of purified water. Throughout the experiment, all rats were unfettered access to water and food and were weighed every 7 days. On the 28th day, 2 h after the administration, the rats were anesthetized intraperitoneally with sodium pentobarbital, blood were collected from the abdominal aorta to prepare serum samples, and liver tissues were dissected and collected. The study protocol was carried out under the approval and supervision of the Center for Experimental Animal Welfare and Ethics of Beijing University of Traditional Chinese Medicine.

### Serum Biochemical Analysis

Blood from rats was collected in 5 ml Vacutainer tubes and then centrifuged for 15 min (1,500 g, 4°C) to collect the upper serum layer as serum samples. Liver injury was assessed by measuring AST, ALT, ALP, DBIL, and TBIL in each rat serum sample using CX4 Pro automated biochemical analyzer (Beckman, Brea, CA, United States) according to the manufacturer’s instructions.

### Organ Weight and Histopathological Assessment

After dissection, the livers and brains of the rats were immediately harvested and weighed after washing with normal saline and wiping dry. Part of rat liver tissue was fixed with 10% neutral formalin for 48–72 h, dehydrated, embedded in paraffin, sectioned, stained with hematoxylin and eosin (H&E), and the pathological changes of liver tissues were observed under the microscope. The remaining livers were kept at −80°C for metabolomics, western blot, MDA, and SOD analysis.

### Preparation of Liver and Serum Metabolisms Samples

In addition to using for histopathological assessment, the rest of the liver tissues were immediately frozen in liquid nitrogen and stored at −80 °C. For UPLC-Q-TOF/MS analysis, liver tissues were cut on dry ice, and 100 mg were weighed precisely into Eppendorf tubes (2 ml) with 1 ml of pre-chilled methanol: acetonitrile: water (2:2:1, v/v), which were homogenized and broken by a homogenizer. The homogenate was centrifuged for 15 min (14,000 g, 4°C), and 900 µl of supernatant from each tube was placed in a new Eppendorf tube and dried in a vacuum centrifuge as the test sample. The samples were redissolved in 100 μl of acetonitrile/water (1:1, v/v) solvent for UPLC-Q-TOF/MS analysis.

The rest of the serum samples were aspirated and placed in Eppendorf tubes (2 ml) frozen in liquid nitrogen and stored at −80°C. For UPLC-Q-TOF/MS analysis, serum samples were slowly thawed at 4°C, and 100 µl aliquots were vortexed with 400 µl cold methanol/acetonitrile (1:1, v/v) incubating at −20°C for 30 min to remove proteins. The mixture was centrifuged for 15 min (14,000 g, 4°C), and 400 µl of supernatant from each tube was placed in a new tube and dried under a vacuum. The samples were redissolved in 100 µl of acetonitrile/water (1:1, v/v) solvent for UPLC-Q-TOF/MS analysis.

### UPLC-Q-TOF/MS Analysis

Samples were analyzed using UPLC (1,290 Infinity LC, Agilent Technologies) coupled with quadrupole time-of-flight (AB Sciex Triple TOF 6600). For HILIC separation, a 2.1 mm × 100 mm ACQUIY UPLC BEH 1.7 µm column (Waters, Ireland) was used. In the positive and negative ESI modes, the mobile phases contained 25 mM ammonium acetate and 25 mM ammonium hydroxide aqueous solution (A) and acetonitrile (B). The gradient elution procedure was as follows (time/B): 0–0.5 min, 95%; 0.5–7 min, 95%–65%; 7–8 min, 65%–40%; 8–9 min, 40%; 9–9.1 min, 40%–95%; 9.1–12 min, 95%. The samples were placed in a 4°C autosampler throughout the analysis. To avoid the effects of fluctuations in the instrument detection signal, samples were analyzed continuously in random order. QC samples were inserted in the sample queue to monitor and evaluate the stability of the system and the reliability of the experimental data. The gradient flow rate was 0.3 ml/min, and the column temperature was kept constant at 25°C. A 2 µl aliquot of each sample was injected.

For mass spectrum (MS) analysis, the AB Triple TOF 6600 mass spectrometer was used for the acquisition of primary and secondary spectra of the samples. The ESI source conditions after BEH Amide chromatographic separation were as follows: Ion Source Gas1 (Gas1): 60, Ion Source Gas2 (Gas2). 60, Curtain gas (CUR): 30, source temperature: 600°C, IonSapary Voltage Floating (ISVF) ± 5500 V (positive and negative modes); TOF MS scan m/z range: 60–1,000 Da, product ion scan m/z range: 60–1,000 Da, product ion scan m/z range: 25–1,000 Da, TOF MS scan accumulation time 0.20 s/spectra, product ion scan accumulation time 0.05 s/spectra; secondary mass spectra were obtained using information. The secondary mass spectra were acquired using information-dependent acquisition (IDA) and high sensitivity mode, Declustering potential (DP): ±60 V (positive and negative modes), Collision Energy: 35 ± 15 eV, IDA settings as follows Exclude isotopes within 4 Da, Candidate ions to monitor per cycle: 10.

### Data Extraction and Multivariate Analysis

Raw data of UPLC-Q-TOF/MS in Wiff format were converted to mzXML format using ProteoWizard, and then XCMS software was used for peak alignment, retention time correction, and extraction of peak areas. In the extracted ion features, only those with more than 50% of non-zero measurements were retained. Compound identification of metabolites was performed by comparing accurate m/z values (<10 ppm) and MS/MS spectra with an in-house database built from available authentic standards.

After normalizing the processed data to the total peak intensity, preto-scale principal component analysis (PCA) and orthogonal partial least squares discriminant analysis (OPLS-DA) multivariate data analysis were performed using the R package (ropls) (Thévenot et al., 2015).

### Metabolic Pathways Analysis and Metabolic Fingerprints Identification

Metabolites obtained from metabolome analysis, with VIP >1 in the OPLS-DA analysis, were further applied to Student’s t-test. Metabolites, that *p*-value < 0.05 and VIP >1, were selected as candidate biomarkers. Pathway analysis of candidate biomarkers was performed using MetaboAnalyst 5.0. The predictive performance of potential biomarkers for liver toxicity was analyzed using receiver operating characteristic curves (ROC) to establish metabolic fingerprints.

### Western Blotting, MDA, and SOD Analysis

Liver tissue, which was kept at −80°C for western blotting, was cut on dry ice, an appropriate amount of liver tissue was weighed, RIPA protein extract was added, and then fully disrupted with a tissue homogenizer, and then placed on ice for 10 min. Then the supernatant was collected by centrifugation (14,000 g, 4°C) for 30 min. The protein concentration was assessed by using the BCA protein assay kit. Electrophoresis was performed by using 10% sodium dodecyl sulphate polyacrylamide gel electrophoresis (SDS-PAGE) to separate the proteins. After electrophoresis, proteins were transferred onto polyvinylidene fluoride (PVDF) membranes using a semi-dry method. The PVDF membranes were immersed in 5% TBST skim milk powder solution for 60 min at room temperature for non-specific closure. Then the PVDF membranes were then incubated for 12 h at 4 °C with the following primary antibodies: anti-GPX4 antibody (ab125066, Abcam, Cambridge, UK; 1:20,000), anti-ACSL4 antibody (ab155282, Abcam; 1:10,000), anti-System x_c_^−^ antibody (ab175186, Abcam; 1:20,000), anti GAPDH antibody (ab9485, Abcam; 1:5,000). After the primary antibody incubation was completed, the PVDF membrane was washed 3 times with TBST, then incubated with hrp-labeled secondary antibody for 1 h at room temperature, and then the PVDF membrane was washed three times with TBST, 5 min each time. Add ECL-plus chemistry and incubate. The film was exposed using an Amersham Imager 680 blot and gel imager, and the grayscale values of the protein bands were analyzed. MDA content in rat liver tissue was assayed according to the manufacturer’s instructions, as well as SOD activity.

### Statistical Analysis

Experimental data were expressed as mean ± standard deviation (SD), and statistical analysis of data was performed using GraphPad Prism software (version 8.0). *p*-values of <0.05 (*), ≤0.01 (**) were adopted for statistical significance. Bodyweight, liver weight, liver/brain weight ratio, liver/body weight ratio, AST, ALP, TBIL, SOD, MDA, and Western blot data were analyzed using Student’s t-test. All mass spectrometry data were processed by XCMS for peak alignment, retention time correction, and extraction of peak areas. The intensity of each ion was normalized according to the total ion count. PCA and OPLS-DA were performed using the R package (ropls). Pathway analysis was performed based on MetaboAnalyst 5.0.

## Results

### Major Compounds Concentrations Analysis

**FIGURE 1:**
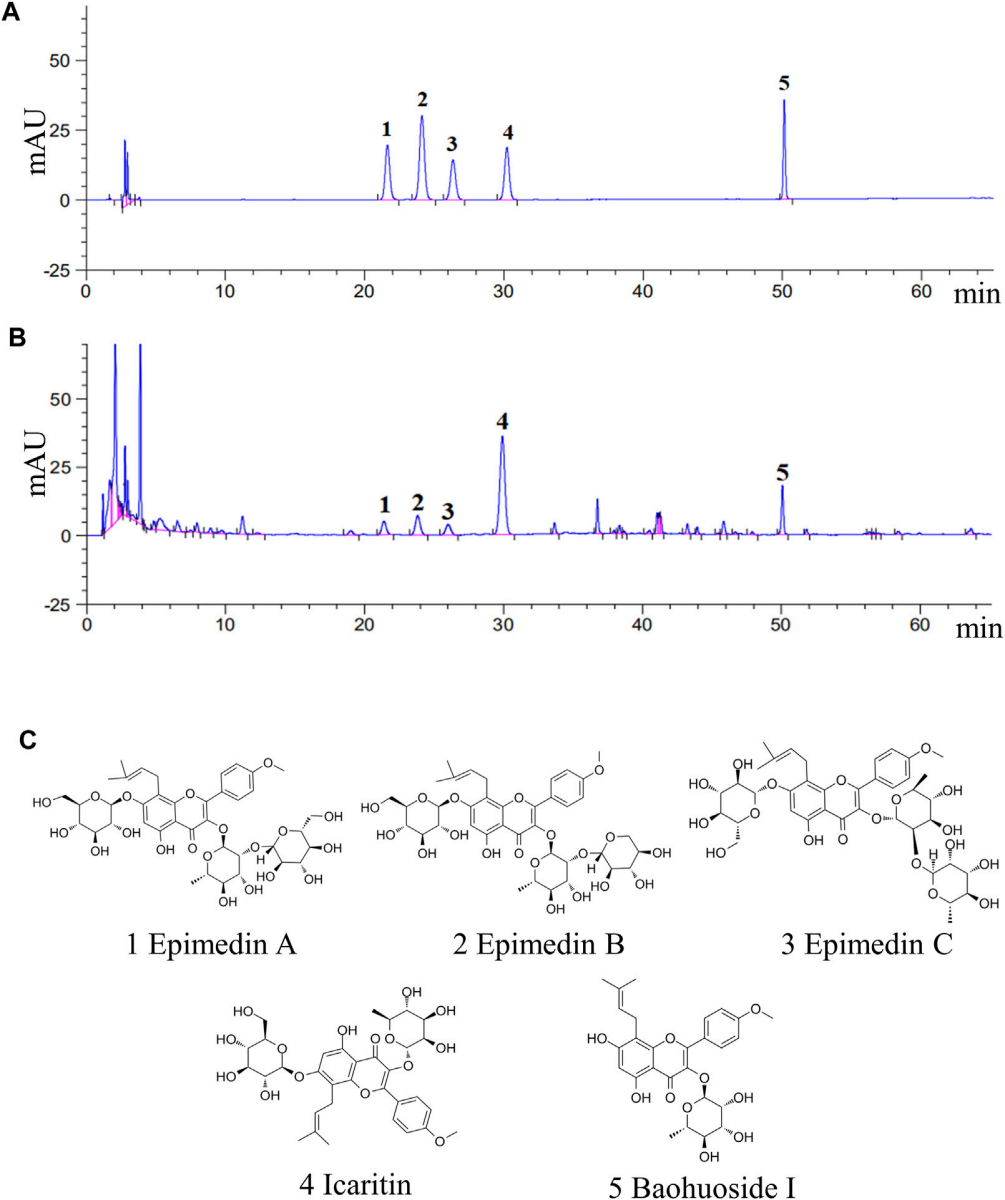
The five main compounds in EEE were quantified by HPLC analysis. **(A,B)** HPLC analysis chromatograms of the 5 main components and EEE samples. 1, epimedin A (C_40_H_52_O_19_); 2, epimedin B(C_38_H_48_O_19_); 3, epimedin C(C_39_H_50_O_19_); 4, icaritin (C_21_H_20_O_6_); 5, baohuoside I(C_27_H_30_O_10_). **(C)** Molecular structures of the 5 main components in EEE.

To ensure the reproducibility and accuracy of the experimental procedure, it is important to evaluate the quality of EEE. Five major components (epimedin A, epimedin B, epimedin C, icaritin, and baohuoside I) in EEE were selected as quality control indicators, and the concentrations of these key compounds in EEE were determined by HPLC. The concentrations of epimedin A, epimedin B, epimedin C, icaritin, and baohuoside I in EEE were 4.5, 7.3, 4.3, 31.8, 6.8 mg/g, respectively. The results are presented in Figure 1.

### Toxic Performances of EEE-Induced Liver Injury in Rats

**FIGURE 2:**
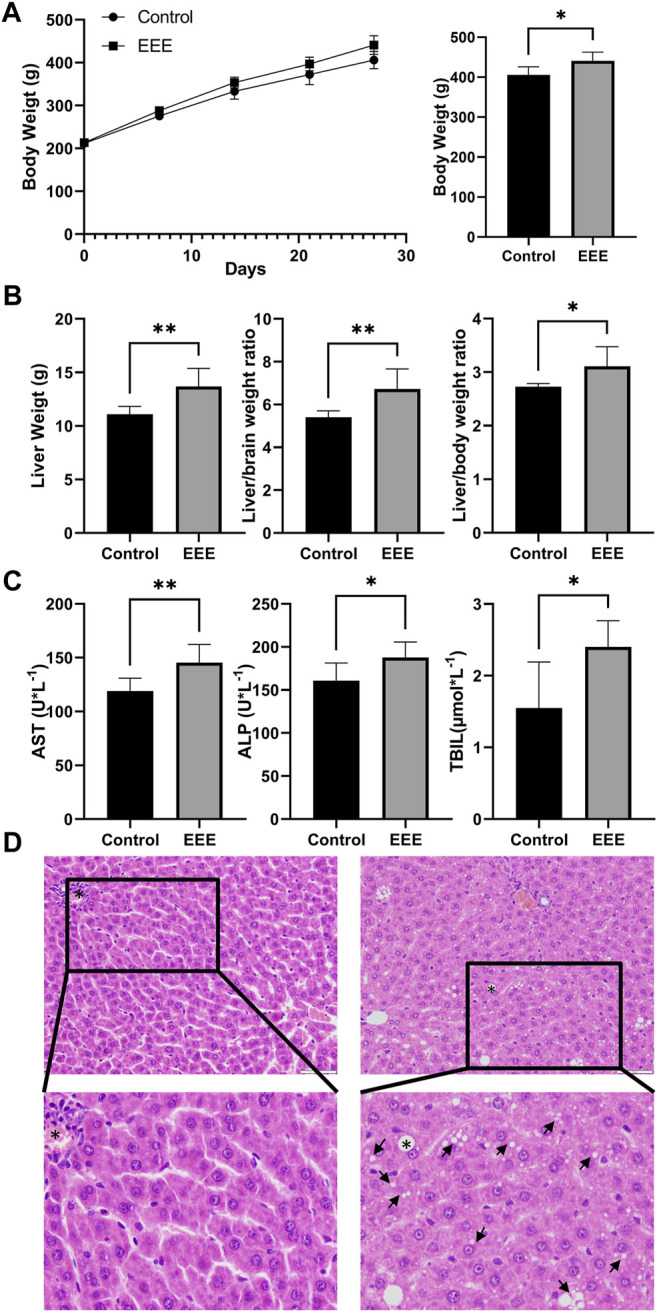
The phenotype of EEE-induced liver injury in rats. **(A)** The EEE-induced phenotype of rat liver injury. The rats were randomly divided into the following two groups: a normal control group of rats (Control); a group of rats exposed to EEE (EEE). Results are presented as mean ± SD in rats, and significant differences are indicated (∗*P*< 0.05, ∗∗*P*≤ 0.01 vs. Control group, *n* = 6). **(A)** Bodyweight of rats during EEE exposure. **(B)** liver weight, liver/brain weight ratio, and liver/body weight ratio after 28 days of exposure. **(C)** Serum levels of AST, ALP, and TBIL were determined after 28 days of exposure. **(D)** Histopathological damage of rat liver was assessed by HE staining (H&E staining, ×200 magnification).

In this study, the body weight, organ weights, serum biochemical parameters, and liver pathology of rats after 28 days of exposure were analyzed to clarify the hepatotoxicity of EEE. Our results showed a gradual increase in body weight of rats in the control and EEE groups during the dosing period; after 28 days of exposure, the body weight of rats in the EEE group was significantly higher than that in the control group (*p* = 0.0157) (Figure 2A). The liver weight, liver/brain weight ratio, and liver/body weight ratio of rats were significantly higher after EEE exposure compared with the control group (*p* = 0.006, 0.008, and 0.0322) (Figure 2B). The results of serum biochemical indexes showed that AST, ALP, and TBIL were significantly higher in rats after treatment with EEE (*p* = 0.01, 0.0345, and 0.018) (Figure 2C). H&E staining analysis of liver sections showed no significant histopathological damage in the control group (Figure 2D). In the EEE group, liver sections showed significant histopathological changes, including visible swelling, hepatocyte steatosis, and severe cytoplasmic vacuolation (Figure 2D).

### Liver and Serum Metabolomic Profile Analysis of EEE-Induced Liver Injury

**FIGURE 3:**
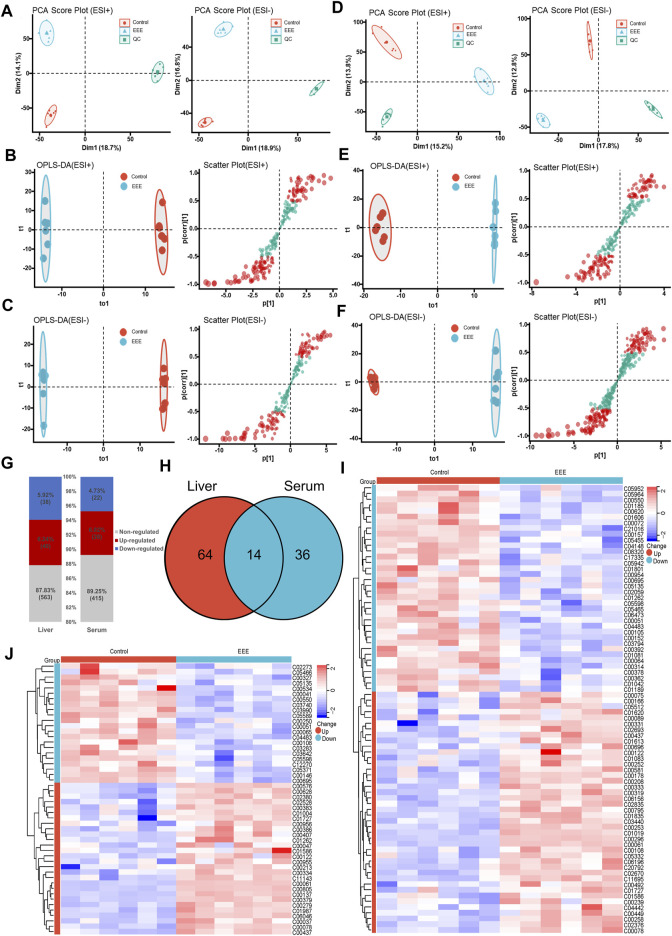
Metabolomic analysis of rats after EEE exposure. **(A–C)** Metabolomic analysis of rat liver tissues. PCA scores **(A)**, OPLS-DA scores **(B)**, and S-plot **(C)** of different groups in liver tissues under positive and negative ESI mode. **(D–F)** Metabolomic analysis of rat serum. PCA scores **(D)**, OPLS-DA scores **(E)**, and S-plot **(F)** of different groups in serum with positive and negative ESI patterns. **(G)** Liver and serum metabolite profiles between normal and EEE rats. **(H)** Shared and unique amounts of liver and serum metabolites were also visualized in the Venn diagram between normal and EEE group rats. **(I)** Heat map of 78 metabolites significantly altered in the liver, clustered in the normal EEE group. The colors from blue to red indicate the relative amounts of metabolites. **(J)** Heat map of 50 metabolites significantly altered in serum, clustered in the normal, EEE group. The colors from blue to red indicate the relative content of metabolites.

The overall metabolic profiles of liver tissues and serum samples in the control and EEE groups were obtained by UPLC-Q-TOF/MS in positive and negative ionization modes, respectively. The score plots of PCA analysis based on the data in positive and negative ion mode were shown in Figures 3A–F. The QC samples were tightly aggregated in the scoring plots for liver, serum positive ion, and negative ion modes, which indicated the stability of the UPLC-Q-TOF/MS system throughout the analysis process and ensured the reliability and accuracy of the results. In the PCA model of liver and serum positive and negative ion assay results, a clear trend of separation was shown between the control and EEE groups (Figures 3A,D). This indicated that the liver and serum metabolic profiles of rats in the EEE administration group were quite different from the control group.

For further screening of differential metabolites in the liver and serum of rats after EEE exposure, the data were first analyzed by applying supervised statistical methods of OPLS-DA and S-plot. The OPLS-DA results (Figures 3B,C,E,F) showed significant separation between liver tissue and serum in the control and EEE groups. In the S-plots (Figures 3B,C,E,F), metabolites were considered to make a significant contribution to the clustering and identification between two groups when the variable VIP value ≥1 and |*P* (corr)| value ≥0.5. Then the metabolomics data were further analyzed by using the student’s t-test. Liver and serum differential metabolites that changed significantly between the control and EEE groups were screened according to VIP >1 and *P*< 0.05. The previously screened differential metabolites were identified by accurate mass-to-charge ratios, and the true mass error was limited to 10 ppm. The structures of the identified metabolites were analyzed and validated, and exogenous metabolites such as pharmaceuticals were removed. Finally, 78 endogenous differential metabolites were obtained in the liver, of which 40 were up-regulated and 38 down-regulated. 50 endogenous differential metabolites were obtained in serum, of which 28 were up-regulated, and 22 were down-regulated (Figure 3G). The m/z, retention time, and structural formulae of liver and serum differential metabolites were listed in Supplementary Tables S1 and S2. Cluster analysis and heatmap were performed for 78 liver differential metabolites, 50 serum differential metabolites, respectively. The results showed that the 78 liver differential metabolites and 50 serum differential metabolites could effectively distinguish the control group from the EEE group (Figures 3I, J).

### The Metabolite Fingerprint of EEE-Induced Liver Injury

**FIGURE 4:**
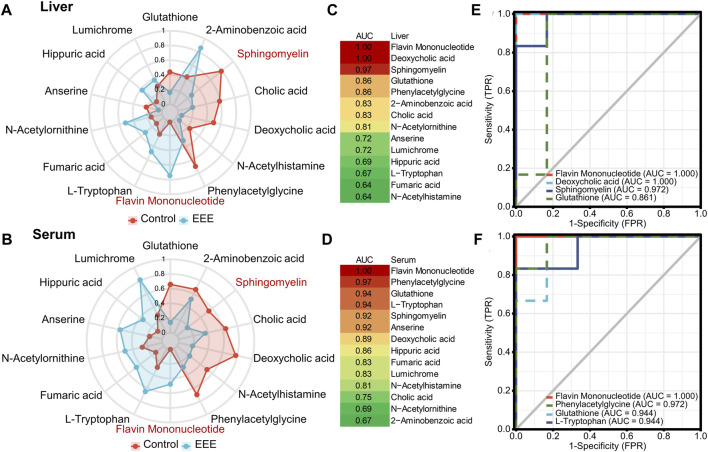
Screening for biomarkers associated with EEE-induced liver injury. **(A,B)** Spider plots of 14 liver and serum shared differentially expressed metabolites between the Control and EEE groups. The red line represents the control group, and the blue line represents the EEE group. **(C,D)** Heat map of AUC values of 14 liver and serum shared differentially expressed metabolites. **(E,F)** ROC curves of differentially expressed metabolites with top 4 AUC values in liver and serum.

Herb-induced liver injury is insidious, so screening for appropriate serum metabolic fingerprints can help establish an early warning method to avoid liver injury. It was found that 14 differential metabolites were significantly different in both liver and serum after EEE exposure (Figure 3H). Among the 14 shared differential metabolites, 12 showed consistent changes in liver and serum (Figures 4A,B). Among them, glutathione (GSH), sphingomyelin, cholic acid, deoxycholic acid, N-acetylhistamine, and phenylacetylglycine were significantly decreased in liver and serum after EEE exposure. Flavin mononucleotide (FMN), L-tryptophan, fumaric acid, N-acetylornithine, hippuric acid, and lumichrome were significantly increased in liver and serum after EEE exposure. ROC curves were used to analyze the predictive performance of 14 differential metabolites for liver injury, and the top 4 differential metabolites with AUC values in liver and serum were selected to plot ROC curves (Figures 4C–F). In the liver, three metabolites’ AUC values > 0.9, including FMN, deoxycholic acid, and sphingomyelin. In serum, six metabolites’ AUC values > 0.9, including FMN, phenylacetylglycine, GSH, L-tryptophan, sphingomyelin, and anserine. Among them, the AUC values of FMN and sphingomyelin were greater than 0.9 in both liver and serum, which could be used as potential biomarkers of EEE hepatotoxicity.

### Pathway Enrichment and Mechanisms Analysis

**FIGURE 5:**
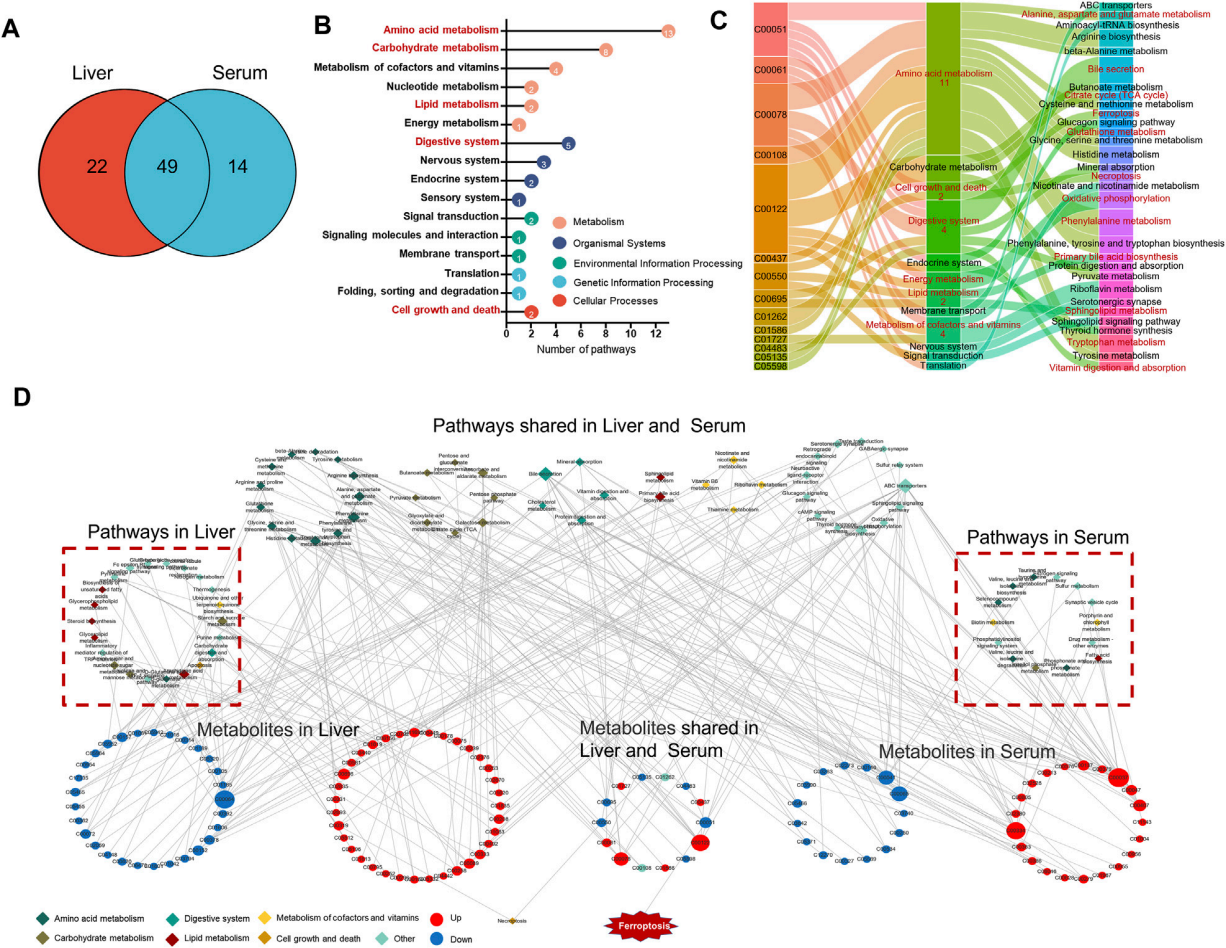
The metabolic pathways and metabolism-related networks of the differential metabolites. **(A)** Venn diagram showing the number of shared and unique pathways for liver and serum differential metabolites. **(B)** Cleveland dot plots show the categories of 49 metabolic pathways shared by liver and serum. **(C)** Sankey diagrams show the relationship of 14 metabolites shared by the liver and serum with key metabolic pathways. **(D)** Network of liver and serum differential metabolites in relation to the KEGG pathway.

To further analyze the effect of EEE exposure on metabolic pathways, the obtained differential metabolites in liver and serum were submitted into MetaboAnalyst 5.0 for KEGG pathway analysis, and the results revealed that the differential metabolites in liver and serum were respectively enriched to 71 and 64 pathways, of which 49 pathways were shared (Figure 5A). According to the KEGG pathway classification, 49 shared pathways are mainly involved in amino acid metabolism, lipid metabolism, digestive system, carbohydrate metabolism, metabolism of cofactors and vitamins, cell growth and death, etc. (Figure 5B). The disturbances of alanine, aspartate and glutamate metabolism, phenylalanine metabolism, arginine biosynthesis, tryptophan metabolism, and glutathione metabolism were observed in amino acid metabolism, disturbances in primary bile acid biosynthesis and sphingolipid metabolism were observed in lipid metabolism, and the disturbances in tricarboxylic citrate cycle, pentose phosphate pathway, and pyruvate metabolism were observed in carbohydrate metabolism. For the digestive system, disturbances in bile secretion, cholesterol metabolism, and other pathways were observed. In addition to the disruptions of thiamine metabolism, riboflavin metabolism, vitamin B6 metabolism, and ferroptosis and necroptosis pathways were observed (Figure 5C).

The KEGG enrichment results were imported into Cytoscape software to construct the KEGG pathways-metabolites network (Figure 5D), it was found that EEE could broadly affect amino acid and lipid metabolism in rats, mainly including primary bile acid biosynthesis, sphingolipid metabolism, bile secretion, cholesterol metabolism, and glutathione metabolism. These pathways are mainly involved in the metabolism of glutathione and the biosynthesis of unsaturated fatty acids. Among these pathways, studies have shown that glutathione metabolism, cholesterol metabolism, and sphingolipid metabolism are closely related to ferroptosis (Agmon and Stockwell, 2017; Stockwell et al., 2020; Liu et al., 2021). Also, the key differential metabolites of GSH, FMN, fumaric acid, etc. in these pathways have important roles in ferroptosis (Ursini and Maiorino, 2020; Wang H et al., 2020; Vabulas, 2021). Combining the above metabolic pathway analysis, the hypothesis was proposed that EEE caused liver injury by inducing ferroptosis.

### EEE-Induced Liver Injury Associated With Ferroptosis

**FIGURE 6:**
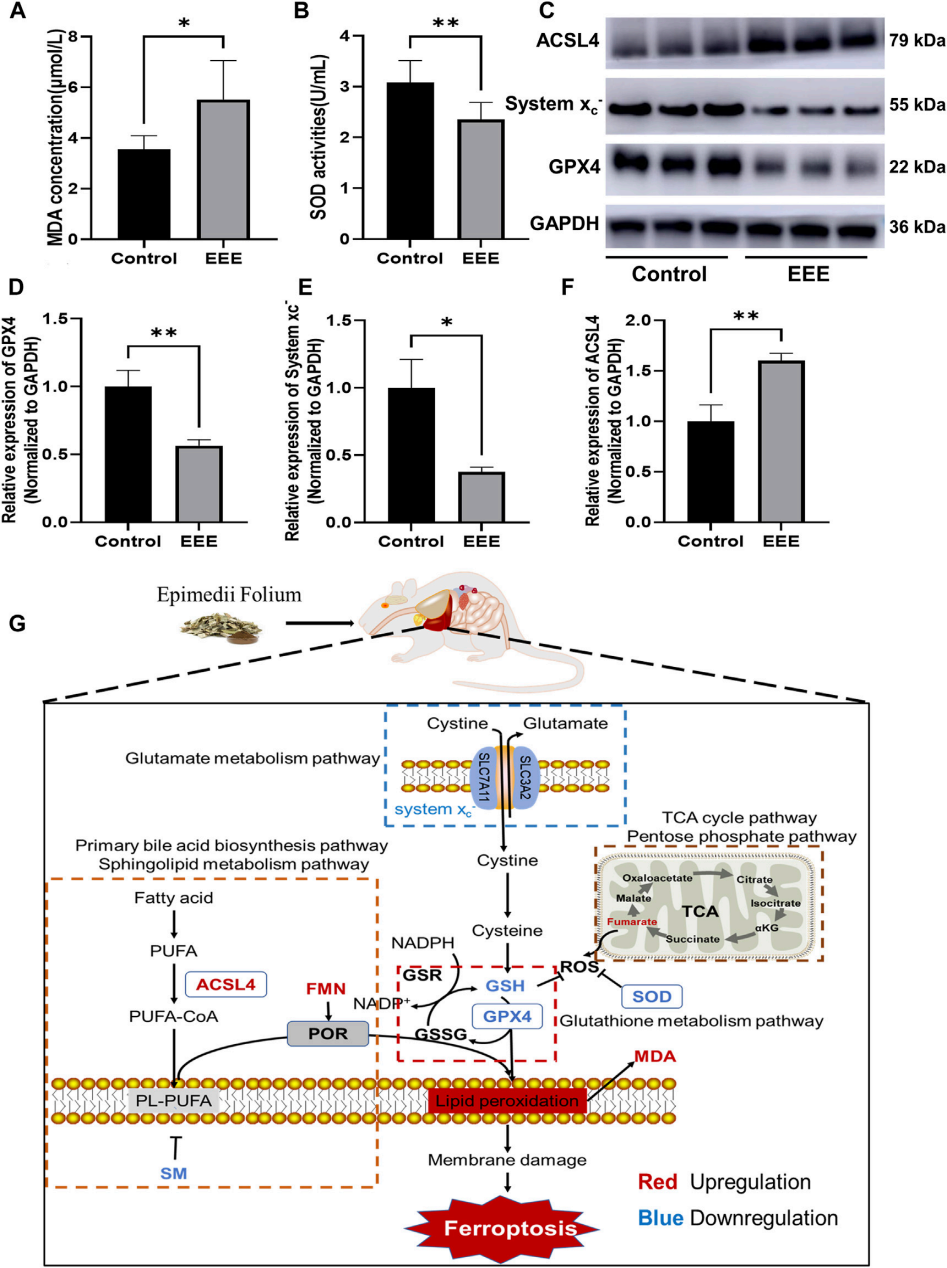
Changes of ferroptosis markers in rat liver after EEE exposure. **(A)** The MDA contents were significantly higher than in the control group. **(B)** The SOD activity was significantly lower than the control group. **(C)** The protein content of GPX4, System x_c_^−,^ and ACSL4 in the liver was detected by immunoblotting. Results are presented as mean ± SD in rats, and significant differences are indicated (∗*p*< 0.05, ∗∗*p*< 0.01 vs. Control group, *n* = 6). **(D)** After EEE exposure, the expression of GPX4 protein was significantly lower than in the control group. **(E)** After EEE exposure, the expression of System x_c_^−^ protein was significantly lower than in the control group. **(F)** After EEE exposure, the expression of ACSL4 protein was significantly higher than in the control group. **(G)** Mechanism of EEE-induced liver injury by triggering ferroptosis based on metabolomic analysis.

To verify whether EEE causes liver injury by inducing ferroptosis in the liver of rats, the typical indicators related to ferroptosis were examined, including the lipid peroxidation marker (MDA), intracellular major antioxidant activity enzyme (SOD), ferroptosis inhibitory protein (GPX4), System x_c_^−^, ferroptosis-promoting protein (ACSL4). The results showed that EEE exposure could significantly increase MDA content and significantly lower SOD activity in rat liver (*P* = 0.0147, 0.0084) (Figures 6A,B). In addition, the results of the Western blot assay showed that EEE exposure significantly decreased the protein expression of glutathione peroxidase 4 (GPX4), System x_c_^−^, while the expression of ACSL4 protein significantly increased (*P* = 0.0066, 0.0155, 0.0071) (Figures 6C–F). Taken together, these results supported that liver injury in rats after EEE exposure could be strongly associated with ferroptosis.

## Discussion

The factors contributing to liver toxicity with herbal medicines are complex, and studying their mechanisms is a great challenge. Liver plays a crucial role in the metabolism, detoxification, and excretion of exogenous chemicals (Sun et al., 2021). When herbal medicines enter the body, they undergo metabolic reactions, and endogenous small molecules change over time as they are metabolized, and these changes in endogenous small molecules may induce liver injury (Liu et al., 2016). Metabolomics is used to characterize changes in the organism in response to external factors by detecting small molecules at the most downstream level of the system biology (Cuykx et al., 2018). This makes metabolomics fingerprinting very sensitive, and even small external factors may induce changes. Metabolomics allows systematic filtering of the metabolic change patterns of the body through the analysis of endogenous metabolites (Wang et al., 2011). And metabolomics helps to identify potential biomarkers and disordered metabolic pathways by comparing metabolic profiles in normal and toxic states, thus elucidating possible mechanisms (Chen et al., 2020). In this study, the hepatotoxicity of EEE in rats was evaluated, and the relevant mechanisms were explored.

Our results showed that the liver weight, liver/body weight ratio, and liver/brain weight ratio of EEE-exposed rats were significantly higher than those of the control group (Figures 2A,B). This demonstrated the toxic effects of EEE on the liver. Serum biochemical parameters are the most commonly used markers of liver function (Green and Flamm, 2002). In our experiments, AST, ALP, and TBIL in the serum of rats exposed to EEE were significantly altered compared with control rats (Figure 2C). Pathology of the liver by microscopy revealed severe cytoplasmic vacuolation in liver cells of rats in the EEE group. Organ weight is one of the most sensitive toxicity indicators, and changes are often earlier or more severe than changes in histopathology or serum indicators (Piao et al., 2013); our results showed that the differences in the indicators such as liver weight, liver/body weight ratio, and liver/brain weight ratio of rats were more obvious than serum biochemical indicators such as AST, ALP, this suggested that the effects of EEE on the liver itself predate the changes in blood biochemistry. These results suggested that EEE might have caused significant damage to the rat liver; how EEE induces these pathological changes requires further investigation.

A metabolomic approach was applied to explore the mechanism of EEE-induced liver injury. The results revealed that EEE exposure led to extensive differences in the metabolism of serum and liver in rats. In-depth analysis revealed that 14 identical endogenous metabolites in serum and liver were significantly different from the normal group after EEE exposure and could serve as metabolic fingerprints for EEE-induced liver injury. Among them, FMN and sphingomyelin had AUC values greater than 0.9 in both liver and serum by ROC analysis and could be used to predict hepatotoxicity of EEE. And the subsequent analysis showed that FMN and sphingomyelin had an important role in the mechanism of EEE-induced liver injury.

The KEGG pathway enrichment analysis was performed on differential metabolites to deeply analyze the mechanism of liver injury caused by EEE. The enrichment results showed that EEE exposure had a great effect on amino acid metabolism, lipid metabolism pathways, especially on primary bile acid biosynthesis, sphingolipid metabolism, bile secretion, cholesterol metabolism, glutathione metabolism, and other pathways related to glutathione metabolism and unsaturated fatty acid biosynthesis metabolism have greater interference (Figures 5A–C). Extensive studies have shown that unsaturated fatty acid peroxidation due to abnormal glutathione metabolism is one of the typical features of ferroptosis (Xie et al., 2016; Doll et al., 2019; Li et al., 2020) (Figure 5D). Ferroptosis is a new type of cell death discovered in recent years, and the process of cell death is usually accompanied by massive iron accumulation and lipid peroxidation (Chen et al., 2021). Increased lipid peroxide content is an important marker for the development of ferroptosis. MDA is one of the degradation products of polyunsaturated fatty acid peroxides, and MDA increases significantly when ferroptosis occurs (Tang et al., 2021). Superoxide dismutase (SOD) is widely found in human tissues and is an important antioxidant that plays an important role in maintaining the redox balance of cells (Rosa et al., 2021). Our previous study found that EEE significantly enhanced reactive oxygen species (ROS) levels, decreased GSH levels, and promoted MDA production in HL7702 and HepG2 cells *in vitro* (Zhang L et al., 2020). Based on these results, we hypothesize that ferroptosis may be the key to EEE-induced liver injury.

The related markers were analyzed to confirm the conjecture that EEE causes ferroptosis; the results of metabolomic showed that GSH in the liver and serum of rats was significantly reduced after EEE exposure (Figures 4A,B). Meanwhile, the SOD activity in liver tissues was significantly decreased, and the level of MDA was significantly increased (Figures 6A,B). Among them, the changes in GSH and MDA were consistent with the results of our previously published *in vitro* experiments. In addition, the metabolomic analysis revealed significantly higher levels of fumaric acid in liver tissue and serum of rats in the EEE group (Figures 4A,B). It has been found that glutathione succinate (GSF), the covalently bound product of fumaric acid and glutathione, is a substrate for glutathione reductase and can enhance ROS production by consuming NADPH(Sullivan et al., 2013). This finding is consistent with the *in vitro* results that ROS levels were significantly increased in HL7702 and HepG2 cells after EEE incubation. In conclusion, the above results demonstrated that EEE induced ferroptosis in rat liver by disrupting redox balance.

Ferroptosis initiation and execution are tightly controlled by iron, lipid, amino acid, and glutathione metabolism; the results of metabolomic showed that amino acid and glutathione metabolic pathways were extensively affected in rat liver after EEE exposure (Figure 5B), the cystine/glutamate transporter (System x_c_^−^) is involved in this procedure and plays an important part in the process (Koppula et al., 2018). The System x_c_^−^ is an amino acid antiporter, a transmembrane structure formed by light solute carrier family 7 member 11 and Solute Carrier Family 3 Member 2 (Liu et al., 2020). The System x_c_^−^ is primarily responsible for pumping intracellular glutamate out of the cell in exchange for extracellular cysteine (Koppula et al., 2021). Cystine entering the cell is converted to cysteine, which is then combined with glutamate and glycine to synthesize the endogenous antioxidant GSH (Lu, 2009), GSH is a tripeptide antioxidant and a cofactor of selenium-dependent GPX4 in reducing lipid peroxidation, reduced GSH synthesis can indirectly inactivate GPX4, lead to the bioaccumulation of intracellular ROS and cause lipid peroxidation (Dixon et al., 2012). GPX4, a member of the GPX family, is the only enzyme, which reduces phospholipid hydrogen peroxide and plays an essential role in maintaining redox homeostasis in cells, GPX4 limits the propagation of lipid peroxidation in membranes by reducing toxic lipid peroxides (L-OOH) to non-toxic lipid alcohols (L-OH), which in turn prevents ferroptosis (Forcina and Dixon, 2019). Thus, ferroptosis is irreversible when oxidative damage leads to a large production of lipid peroxides or when excessive depletion of GSH leads to a decrease in GPX4 activity (Yang and Stockwell, 2016). Recent studies have shown that inhibition of the System x_c_^−^, which reduces the uptake of cystine, impairs the antioxidant defense system of cells and eventually leads to ferroptosis (Dixon et al., 2014). It was also found that overexpression of GPX4 in cells caused resistance to ferroptosis, while knockdown of GPX4 promoted ferroptosis (Bersuker et al., 2019). Therefore, with reduced GPX4, System x_c_^−^ expression is a key marker of ferroptosis. In our experiments, the expression of System x_c_^−^ and GPX4 in rat liver was significantly reduced after EEE exposure (Figures 6D,E), which is one of the pieces of evidence confirming that EEE might induce ferroptosis. Iron-catalyzed excessive peroxidation of phospholipids (PLs) containing polyunsaturated fatty acids (PUFAs) is a major feature of ferroptosis, and these phospholipids are abundant in mammalian cell membranes (Tang et al., 2021).

The results of metabolomic showed that fatty acid metabolism pathways such as the Primary bile acid biosynthesis pathway and sphingolipid metabolism pathway in rat livers were extensively affected after EEE exposure (Figure 5B, Figure 6G). The study revealed through lipidomic analysis that Acyl-CoA Synthetase Long Chain Family Member 4 (ACSL4) could have an essential role in ferroptosis by regulating the metabolism of lipid components (Doll et al., 2017). Several studies have identified ACSL4 as a key factor in determining ferroptosis sensitivity (Yuan et al., 2016; Doll et al., 2017; Kagan et al., 2017). A significant increase in ACSL4 expression in rat liver tissue after exposure to EEE was observed (Figures 6C,F). Therefore, based on the above results, we infer that EEE may induce hepatic lipid metabolism disturbance and promote ferroptosis by upregulating ACSL4.

In addition, interestingly, FMN, sphingomyelin, which had AUC values greater than 0.9 in both liver and serum, also had an important role in ferroptosis. Among them, FMN is a biomolecule produced by riboflavin (vitamin B2) through riboflavin kinase, which is an auxiliary group of various oxidoreductases (such as NADH dehydrogenase) (Akasov et al., 2019). FMN is a more potent oxidant than Nicotinamide adenine dinucleotide (NAD) and enhances the ability of cytochrome P450 oxidoreductase (POR) to auto-oxidize and produce ROS, and promotes PUFAs peroxidation to induce ferroptosis (Esteves et al., 2020; Yan et al., 2021). Sphingolipids are significant components of animal plasma membranes; some studies have found that it may act as a “biophysical antioxidant” by changing the oxidation rate of PUFA, limiting the propagation of the lipid peroxidation process, and thereby reducing ferroptosis (Coliva et al., 2020; Aldrovandi et al., 2021). Our experiments revealed that the content of FMN in the liver and serum was significantly increased, and the content of sphingomyelin was significantly decreased after EEE exposure (Figures 4A,B). These also indicated that EEE exposure resulted in severe redox derangements in rat liver, leading to the development of lipid peroxidation, which in turn caused ferroptosis in hepatocytes.

Some researchers have found that GPX4 and GSH, which have important regulatory roles in ferroptosis, may have inhibitory effects on NLRP3 inflammasome activation (Wang et al., 2019; Zhang et al., 2021). In our study, it was also found that GPX4 and GSH decreased after EEE administration, which could not only induce ferroptosis in liver cells to produce direct toxicity, but also lead to non-specific liver injury by activating the NLRP3 inflammasome. According to literature reports, not only EEE can cause direct liver injury, but its main components such as icariside I and icariside II can also induce idiosyncratic liver injury (IDILI) by activating the NLRP3 inflammasome (Wang Z et al., 2020; Gao et al., 2021). In addition, in the KEGG pathway analysis, we found that metabolic disturbance caused by EEE might also cause necroptosis in hepatocytes. Necroptosis is a way of programmed cell death mediated by inflammation (Dhuriya and Sharma, 2018). NLRP3 inflammasome activation often leads to necroptosis in cells (Huang et al., 2021). In addition, studies have found that GPX4 is not only an inhibitor of ferroptosis, but also plays an important role in inhibiting necroptosis (Canli et al., 2016). At the same time, some studies have found that the redox homeostasis of cells also plays an important role in necroptosis (Florean et al., 2019; Zhang Y et al., 2020; Li et al., 2021). Decreased GSH and SOD levels lead to increased oxidative stress in cells, which can induce necroptosis in cells (Xie et al., 2015). Taken together, EEE exposure caused a severe imbalance in redox homeostasis in the rat organism, induced ferroptosis in hepatocytes, and activated inflammatory pathways promoting necroptosis. As for the relationship between EEE and IDILI, we expected to have some new findings in further research. In combination, we found that the liver injury caused by EEE exposure might be related to the extensive disruption of amino acid metabolism, glutathione metabolism, and lipid metabolism in rat liver, and the regulation of the expression of key ferroptosis proteins such as GPX4, System x_c_^−^, and ACSL4, resulting in the decrease of antioxidant active substances such as GSH, sphingomyelin and SOD, and the increase of pro-oxidant active substances such as FMN and ROS, which disrupt the redox balance in the liver and promote lipid peroxidation (increased MDA content), causing ferroptosis (Figure 6G).

## Conclusion

In conclusion, our study confirmed that EEE could induce liver injury in rats, and its hepatotoxicity mechanism was related to the induction of ferroptosis in hepatocytes. During the performance of metabolic profiling of rat liver and serum, we found the metabolic difference and screened two differential metabolites (FMN, SM) shared by liver and serum as potential biomarkers for EEE-induced liver injury (ROC AUC >0.9). By analyzing the differential metabolites and enrichment pathways, the results suggested that EEE might induce liver injury by disrupting the redox homeostasis of rat liver tissue inducing ferroptosis. This finding was confirmed by the expression of ferroptosis marker proteins GPX4, System x_c_^−^, ACSL4, as well as SOD activity and MDA content in the liver. These results provided a theoretical basis for further research on the mechanism of EEE-induced liver injury. However, there were some limitations in this study. The current experimental results did not fully uncover the mechanism of *Epimedium koreanum* Nakai hepatotoxicity, and the main components responsible for hepatotoxicity had not been clarified, which needed further exploration and research.

## Data Availability

The original contributions presented in the study are included in the article/Supplementary Material; further inquiries can be directed to the corresponding authors.
