# Supplementary material for: Epimedium koreanum Nakai–Induced Liver Injury—A Mechanistic Study Using Untargeted Metabolomics
Source: Front Pharmacol. 2022 Jul 13;13:934057. doi: 10.3389/fphar.2022.934057 (PMC9326364; doi:10.3389/fphar.2022.934057)
Supplement: Supplementary file 3 [file DataSheet1.docx]

Supplementary Material

# Supplementary Figure and Tables

## Supplementary Figures


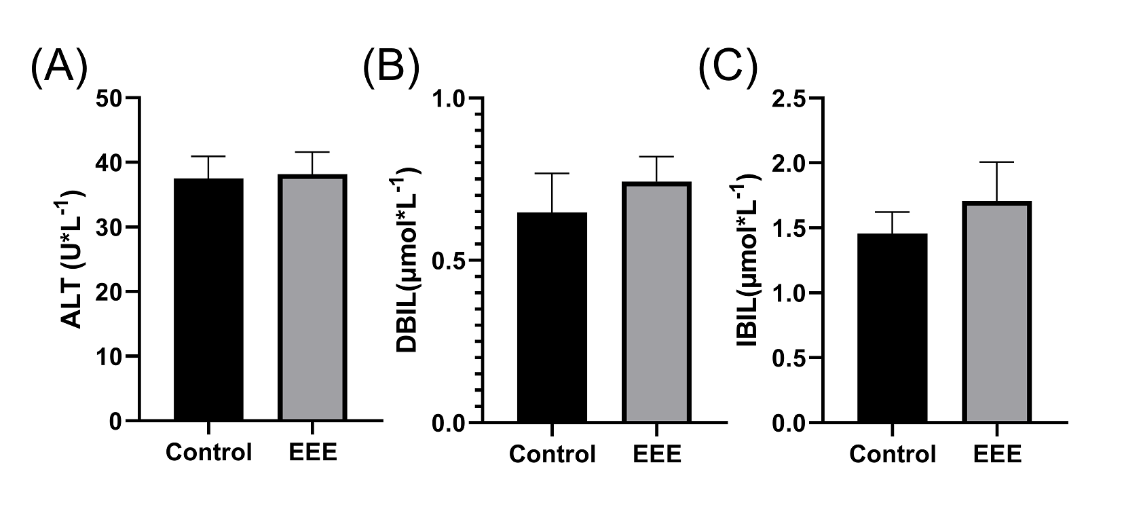


**Supplementary Figure 1.**. Serum levels of ALT, DBIL, and IBIL were determined after 28 days of exposure.

## Supplementary Tables

Supplement Table1 The details of liver differential metabolites

Supplement Table2 The details of serum differential metabolites
